# A simplified Gibson assembly method for site directed mutagenesis by re-use of standard, and entirely complementary, mutagenesis primers

**DOI:** 10.1186/s12896-022-00740-y

**Published:** 2022-03-13

**Authors:** Shunit Olszakier, Shai Berlin

**Affiliations:** Department of Neuroscience, Ruth and Bruce Rappaport Faculty of Medicine, Technion- Israel Institute of Technology, Haifa, Israel

## Abstract

**Background:**

Site-directed mutagenesis (SDM) is a key method in molecular biology; allowing to modify DNA sequences at single base pair resolution. Although many SDM methods have been developed, methods that increase efficiency and versatility of this process remain highly desired.

**Method:**

We present a versatile and simple method to efficiently introduce a variety of mutation schemes using Gibson-assembly but without the need to design uniquely designated Gibson primers. Instead, we explore the re-use of standard SDM primers (completely overlapping in sequence) in combination with regular primers (~ 25 bps long) for amplification of fragments flanking the site of mutagenesis. We further introduce a rapid amplification step of the Gibson-assembled product for analysis and quality control, as well as for ligation, or re-ligation at instances the process fails (avoiding expenditure of added Gibson reaction mixtures).

**Results:**

We first demonstrate that standard SDM primers can be used with the Gibson assembly method and, despite the need for extensive digestion of the DNA past the entire primer sequence, the reaction is attainable within as short as 15 min. We also find that the amount of the assembled Gibson product is too low to be visualized on standard agarose gel. Our added amplification step (by use of the same short primers initially employed) remedies this limitation and allows to resolve whether the desired Gibson-assembled product has been obtained on agarose gel or by sequencing of amplicons. It also provides large amounts of amplicons for subsequent ligations, bypassing the need to re-employ Gibson mixtures. Lastly, we find that our method can easily accommodate SDM primers with degenerate sequences.

**Conclusion:**

We employ our alternative approach to delete, replace, insert, and degenerate sequences within target DNA sequences, specifically DNA sequences that proved very resistant to mutagenesis by multiple other SDM methods (standard and commercial). Importantly, our approach involves the re-use of SDM primers from our primer-inventory. Our scheme thereby reduces the need (and time and money) to design and order new custom Gibson-primers. Together, we provide a simple and versatile protocol that spans only 4 days (including the added amplification step), requires minimal primer sets and provides very high yields and success rates (> 98%).

**Supplementary Information:**

The online version contains supplementary material available at 10.1186/s12896-022-00740-y.

## Introduction

Site-directed mutagenesis (SDM) is a pivotal molecular biology technique for rationally modifying DNA sequences, for instance substitution, deletions or insertions of base pairs (bps) at desired location within a template DNA [1]. There are different approaches to obtain SDM (most are now commercial [2–6]), but they all share two common denominators: a set of complementary primers that bear the desired change in sequence, mainly, at the center of the primer, and a thermocycler (Polymerase Chain Reaction; PCR)-based amplification step. However, and despite broad-usage, SDM methods provide low efficiency in introducing mutations within DNA and, resultantly, multiple trials are often needed to establish optimal settings for the reaction to succeed [7, 8]. The reason for the low efficiency stems from several notable challenges in many steps of the process; from the initial design of the primers (requiring lengthy primer of high melting temperature and high GC content, and these tend to yield primer dimers and/or strong secondary structures), to the PCR reaction itself (very prolonged reactions, very low reaction yields that usually cannot be distinguished on gel, potential occurrence of mutations in large plasmids, high background from *dpnI*-evaded template DNA, etc.). Moreover, the resulting SDM-PCR products (amplicons) are typically ligated within the bacteria, which may result in unwarranted additions or deletions in the plasmid [9]. Lastly, the approach is limited in the size of the insertion or deletion that can be introduced by one pair of primers. Other methods, such as seamless introduction of residues by blunt-end ligations, require costly primers of lengthier production times (due to 5′ phosphate additions), are of lower efficiency and may introduce mutations at the ligation site (see [10, 11]). These acute challenges are the major reasons why most users are deterred from employing SDM, and why new methods are continuously reported [2, 8]. Thus, means to obtain higher efficiency SDM—rapidly and easily with added degrees of versatility—remain warranted.

We sought to develop a rapid, simple, and versatile method to improve efficiency of the process to, ultimately, reduce time to completion. These considerations have led us to test whether we could insert, delete, or substitute DNA sequences, as well as degenerate residues, within target sequences by employing the Gibson assembly method but, importantly, without requiring the design of designated Gibson primers. Briefly, the Gibson assembly approach is intended for assembly of multiple DNA-segments in a *one-tube-reaction* [12]. Prior assembly, each segment is amplified by use of unique primers (i.e., Gibson primers) to introduce a 15–20 nucleotide sequence at both 5′- and 3′-termini and these added sequences serve for complementation and assembly between the amplified fragments. Thereby, Gibson primers are unique to each reaction, often require optimization and are approximately twice longer than standard amplification primers (~ 40 bp vs ~ 20 bp) [13, 14]. Of note, whereas the original report [12] describes the assembly of DNA fragments, it does not detail any mutagenesis schemes. Nevertheless, a few commercial kits have been developed and suggest a mutagenesis scheme via the Gibson assembly (e.g., [15]). However, these do not starkly diverge from the original report, and still require the design of multiple sets of custom and even lengthier Gibson primers. Closer scrutiny of a commercial protocol suggests the design of primers with excessively high melting temperatures (e.g., 70 °C) and strong likelihood of unwarranted (and thereby low yield) PCR products. These demonstrate that the design of these primers is less intuitive and may require added optimization steps that many users, especially non-Gibson experts, will find challenging. Notably, and to the best of our knowledge, degeneration of sequences remains completely unaddressed by recent developments [13, 14] and it remains unknown whether the Gibson assembly could accommodate other *types* of primers. Lastly, in all instances (whether original or subsequent commercial demonstrations) there are no quality control steps to assess whether the reaction (i.e., assembly of fragments) has succeeded. Instead, most troubleshooting steps revolve around the use of larger amounts of amplicons for assembly by the reaction mixture or use of larger amounts of product from the bacteria. We deem these steps as unwarranted because Gibson reaction mixtures are expensive and most kits provide a very limited number of reactions.

**Fig. 1:**
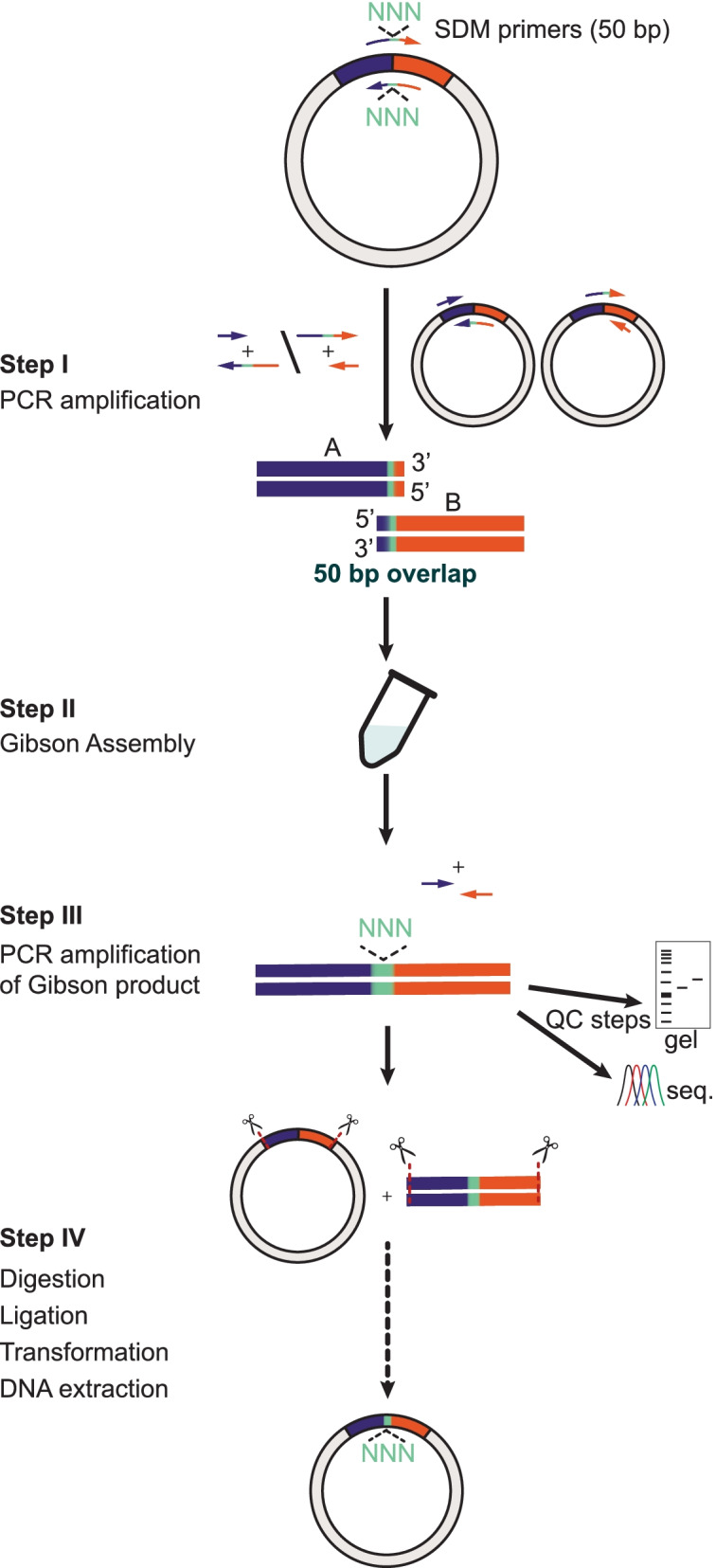
The modified Gibson assembly method using SDM primers and added quality control steps. Flowchart of the method’s main steps. Step I—PCR amplification of the DNA sequence by use of standard SDM primers (50 bp; purple–orange arrows) containing the desired change in sequence, i.e., mutation (green highlight). Each SDM primer (sense, + ; antisense, −) is used separately to amplify the fragments (A and B) flanking the site of mutagenesis (green). This is achieved by the additional use of regular amplification primers (~ 20 bp, short purple and short orange arrows). Step II—A and B amplicons are assembled using the Gibson reaction mixture. Step III—the resulting assembly is amplified by the same primers employed in step I. Note that these primers can only amplify the assembled (correct) fragments. The large amounts of product obtained by this amplification can be used to (1) visualize and examine size of products by electrophoresis (1% agarose gel, cartoon) and (2) products can be isolated and sent to sequencing (cartoon chromatogram). These are the added quality control steps (QC) introduced. Step IV—The amplified assembly product is digested and ligated into a desired plasmid (vector) and transformed into competent cells. The entire process spans 4 days

Here, we present a versatile and simple approach for insertions, deletions, and evolution of DNA sequences by a modified Gibson assembly approach that enables users to re-use standard, non-Gibson, SDM primers extant in their inventory (Fig. 1). We further introduce two quality control steps to increase the success rate of the process and bypass the need for additional assemblies. We show the validity of our approach by insertion, deletion, replacement, and degeneration of residues within target sequences that could not be obtained by standard commercial methods. The entire process spans only 4 days with very high success rates.

## Results

### Introducing six bps into dLight

We have previously designed standard SDM primers for insertion of six bps (TGAATG) at positions 50 or 120, namely prior to the first and second intracellular loops (ILI or ILII, respectively) of a fluorescent dopamine probe (dLight [15]). This insertion should translate into a stop codon followed by a methionine (Stop-Met) in the sequence (Table 1; bold). However, we have repeatedly failed to obtain the desired product by use of the primers with standard approaches or commercial kits (in multiple attempts, see below). In fact, these failures could not be remedied by systematic modifications of annealing temperatures, steps’ durations, number of cycles, added reagents (e.g., DMSO), or change of commercial kits (see Additional file 1: Supplementary text for details). We therefore asked ourselves whether we could obtain these modifications by employing Gibson assembly, but without ordering new primers (longer, more expensive and more time delays), rather by re-use of the existing SDM primers. Of note, at first we were reluctant to do so because SDM primers do not abide to the design rules of Gibson primers. Gibson primers need to contain only partial overlap in sequences between the fragments to be ligated. SDM primers, on the other hand, are not partially, rather completely overlapping. Moreover, is it unknown whether the reaction could engender sufficient digestion of the amplicons to remove matching sequences between the fragments (that would otherwise prevent the fragments from annealing to one another). In fact, the digestion would need to proceed past the entire sequence of the primer to enable ligation (Additional file 2: Fig. S1). Secondly, the reaction might over-digest and produce unwarranted overlaps and incorrect ligations. We suggest that these unknowns are likely the reason why this has never been attempted previously and therefore warranted further scrutiny.

**Table 1:** List of the primers employed in this work

Primer name	Primer sequence (5′–3′)
ILI_SDM_F	GGTCTGTGCTGCCGTTATC**TGAATG**AGGTTCCGACACCTGCGG
ILI_SDM_R	CCGCAGGTGTCGGAACCT**CATTCA**GATAACGGCAGCACAGACC
ILII_SDM_F	CCTCTGTGTGATCAGCGTG**TGAATG**GACAGGTATTGGGCTATCTC
ILII_SDM_R	GAGATAGCCCAATACCTGTC**CATTCA**CACGCTGATCACACAGAGG
ILIII_R	CGTTAATGAGTGAGCTCAGCATTCACTGTTTCTGAGCAATCCTG
hSyn promoter_F	CGCACCACGCGAGGCGCGAGATAGG
FP_R	CTTGTACAGCTCGTCCATGCC
hChR_203R_F	GCATATATCGAGGGTTATCATACT**AGG**GTGCCAAAGGGTCGGTGCCGCCAG
hChR_203R_R	CTGGCGGCACCGACCCTTTGGCAC**CCT**AGTATGATAACCCTCGATATATGC
CAG promoter_F	GCAACGTGCTGGTTATTGTG
hChR_203All_F	GCATATATCGAGGGTTATCATACTTGA**NNN**GTGCCAAAGGGTCGGTGCCG
hChR_203All_R	CGGCACCGACCCTTTGGCAC**NNN**TCAAGTATGATAACCCTCGATATATGC

**Fig. 2:**
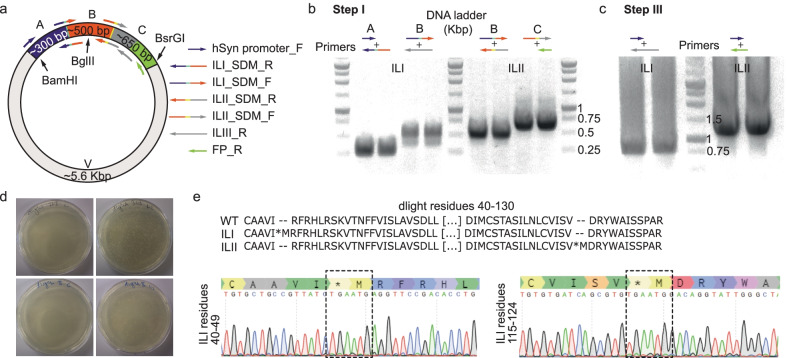
Insertion of six base pairs by the modified Gibson approach into two distinct sites within dLight. **a** Depiction of the dLight-GFP (green fluorescent protein) synthetic gene (colored segments, A–C). Fragment A includes sequences spanning from the promoter of the plasmid and intracellular linker I (ILI; purple); fragment B spans from ILI to ILII (orange); fragment C, ILII to the end of the GFP (green). Backbone of plasmid is light grey. The mutations (cyan or yellow) are situated between the purple and orange fragments, and orange and grey fragments, respectively. Sizes (# of bps) of the fragments are noted within the fragments. Intrinsic and unique digestion sites are also noted (BamHI and BsrGI). Primers used for amplification of each fragment are noted on the right (with corresponding colors). **b** Image of PCR products from step I on 1% agarose gel. DNA ladder sizes (in Kbp) are noted on the right of third ladder. For clarity, we have cropped the images of the gel to show the relevant bands. Full length agarose gels for all figures are provided in Additional file 3: Fig. S5. **c** The assembled Gibson products after amplification by hSyn promoter_F and the ILIII_R primers, and FP_R primers. **d** Image of the agar plates with colonies obtained after ligation of assembled Gibson products to the plasmid (at 1:2 ratio, V:V; top—ILI, bottom—ILII), and controls (left images; backbone plasmid without inserts). **e** Top—Amino acid sequences of *WT*, and expected insertion within ILI and ILII, middle and bottom sequences, respectively. Bottom—The resulting sequences and chromatograms from DNA isolated from colonies (dashed boxes shows the correct modifications)

To test our hypothesis, we first amplified the sequences flanking the site of insertion (sites A and B) by re-using the unsuccessful complementary SDM we had at-hand (see Additional file 1: Supplementary text for details), along general primers from our inventory. Specifically, for amplification of fragment A, we employed the antisense SDM primer (**ILI_SDM_R**) along a sense primer annealing to the promoter of the plasmid (**hSyn promoter_F**) (Fig. 2a, Tables 1, 2). Similarly, amplification of fragment B was obtained by the sense complementary SDM primer (**ILI_SDM_F**) and a standard antisense primer previously used (**ILIII_R**) (Fig. 2a). For an identical insertion at ILII (flanked by sites B and C), we employed the same strategy; combining ILII_SDM_F/R with another general-use primer that anneals to the 3′ terminal of GFP; a sequence that is shared by many other fluorescent proteins (FPs) [16] (Fig. 2a, **FP_R**, green, Tables 1, 2). A standard amplification protocol yielded two sets of products at very high amounts (and rapidly, < 1 h, see Methods; Tables 2, 3) and these could easily be distinguished and assessed on 1% agarose gel (~ 300 and ~ 550 bps, ~ 500 and ~ 650 bps for Parts A, B and C of ILI or ILII, respectively) (Fig. 2b; Please note that image of agarose gel has been cropped for clarity. Images of all full-length gels are provided in the Additional file 3: Fig. S5). To assemble the fragments (A with B, and B with C), we reverted to use the Gibson assembly mix, even though the extent of complementation between our fragments does not meet the requirement of Gibson primers (Additional file 2: Fig. S1) [12]. Having assumed that longer DNA-excision times by the T5-exonuclease would be required to remove the entire sequence of the primer for ligation (Additional file 2: Fig. S1), we placed the isolated PCR products from step I within the Gibson reaction mixture for prolonged incubation (2 hrs; Methods). Notably, the expected product of this assembly (~ 800 or ~ 1150 bps for ILI and ILII, respectively) could not be visualized on 1% agarose gel (see example below in Fig. 4c). We therefore could not determine whether the reaction succeeded and, in case it did not, we could not judge which step was faulty (for instance whether the digestion of the overlapping sequences was insufficient, unattainable or whether excessive digestion has occurred). We therefore opted to try to detect the potential Gibson-ligated product by amplifying it using primers employed in step I, namely sense hSyn promoter_F with the ILIII_R or FP_R antisense primer, for ILI or ILII, respectively. Importantly, we chose these pairs of primers as they can only amplify the ligated product, if extant in the tube. This rapid PCR reaction (1 hr) yielded easily detectable amplicons of the correct size (Fig. 2c, step III; ~ 1 Kbp). Reassured by the latter, we next digested the amplicons and plasmid, ligated them overnight, followed by bacterial transformation and plating (Fig. 2d). We isolated DNA from several colonies and visualized them on 1% agarose gel. Though a handful of colonies did not contain the right plasmid (‘negative’ colonies), all other colonies tested contained the desired substitutions (Fig. 2e). Together, we find that we could easily introduce the desired six bps at two distinct DNA regions—after only 4 days—by a modified Gibson approach employing standard SDM primers.

**Table 2:** Reaction mixture for the KAPA HiFi HotStart ReadyMix PCR Kit

Component	Step I	Step III
2X KAPA HiFi HotStart ReadyMix	12.5 µl	12.5 µl
Template	1 µl (10 ng)	1 µl (Gibson mix)
SDM_Primer_F (10 µM stock)	0.75 µl	0.75 µl
SDM_Primer_R (10 µM stock)	0.75 µl	0.75 µl
ddH_2_O	Up to 25 µl	Up to 25 µl

**Table 3:** PCR settings for KAPA HiFi HotStart ReadyMix PCR Kit

Step	Step I		Step III	
	Cycles	Temperature/duration	Cycles	Temperature/duration
Initial denaturation	1	95 °C/3 min	1	95 °C/3 min
Denaturation	30	98 °C/20 s	30	98 °C/20 s
Annealing		60/56 °C/20 s		60/56 °C/20 s
Extension		72 °C/1 min		72 °C/2 min
Final extension	1	72 °C/5 min	1	72 °C/5 min

### Deletion and replacement of residues in ChR2 using SDM primers

**Fig. 3:**
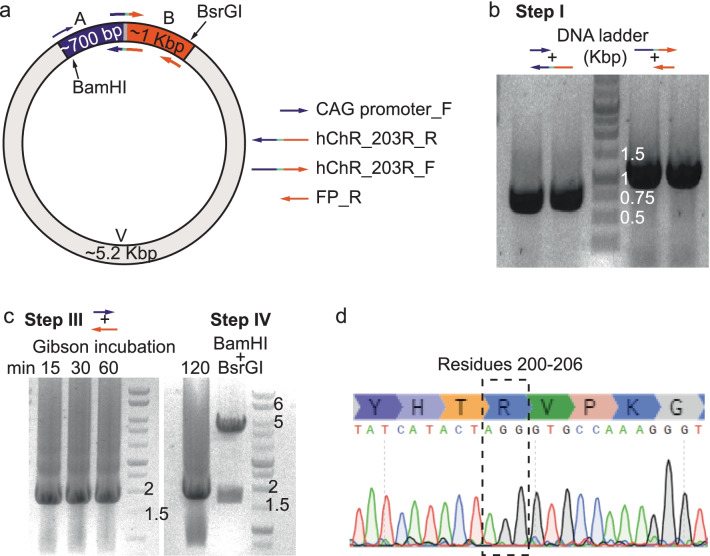
Deletion and replacement of residues within hChR2. **a** Depiction of the hChR2-mCherry synthetic gene (colored segments, A and B). Fragment A includes sequences spanning from the standard promoter of the plasmid and intracellular linker III (ILIII; purple); fragment B spans from ILIII to the end of the mCherry (orange). Backbone of plasmid is light grey. The mutations (cyan) are situated between the purple and orange fragments. Sizes (# of bps) of the fragments are noted within the fragments. Intrinsic and unique digestion sites are indicated (BamHI and BsrGI). Primers used for amplification of each fragment are noted on the right (with corresponding colors). **b** Image of PCR products from step I on 1% agarose gel. DNA ladder sizes (in Kbp) are noted on the right of ladder. **c** The amplified assembled Gibson products, obtained from varying incubation times (15 to 120 min) by CAG promoter_F and the FP_R primers (left), are visualized on 1% agarose gel and analyzed by enzymatic digestion (right panel, BamHI and BsrGI). **d** Sequences, and matching chromatograms, of DNA sequences obtained from resulting colonies (dashed box)

We were next interested in testing whether our modified procedure could support a slightly more challenging procedure, namely to remove and replace six bps by three other (from TGAATG to AGG) at the ILIII (residue 203) of a humanized Channelrhodopsin2-mCherry variant (hChR2-mCherry). We intentionally re-used two standard complementary sets of SDM primers (51 bps each, with 53% GC content) with which we could not obtain the final product under standard SDM conditions (Table 1, and see Additional file 1: Supplementary text). We similarly amplified the two segments of hChR2 using each of the SDM primers in two separate PCR reactions, in combination with a standard sense primer annealing to the promoter (CAG promoter_F), and a general antisense primer annealing to FP (**FP_R**) (Figs. 3a). This reaction yielded the correct amplicons sizing at ~ 700 and ~ 1040 bps (Part A and B, respectively) (Fig. 3b; step I).

Fragments were assembled by the Gibson reaction mixture but, this time around, we varied the incubation times (spanning 15 min to 2 hrs) immediately followed by the second PCR amplification step of the potentially ligated fragment by use of the distal CAG_promoter and FP_R antisense primers (~ 1.5 hr, see Tables 2, 3) (Fig. 3c). Surprisingly, even the shortest Gibson assembly reaction (15 min) yielded the expected ligated product; easily visualized on 1% agarose gel (Fig. 3c, ~ 1.8 Kbp). We then introduced another quality control step consisting of the sequencing of the amplified ligation-product; a procedure that requires sufficiently large amounts of clean product as obtained here. Indeed, we find the desired changes in the DNA the following day in the sequencing results (Additional file 4: Fig. S2). Then, insert and plasmid were digested, ligated, transformed, and plated (Methods). Lastly, we sequenced isolated DNA from only three colonies and find the desired modifications in all three (Fig. 3d).

### Gibson assembly using degenerate primers to evolve a single residue in ChR2

**Fig. 4:**
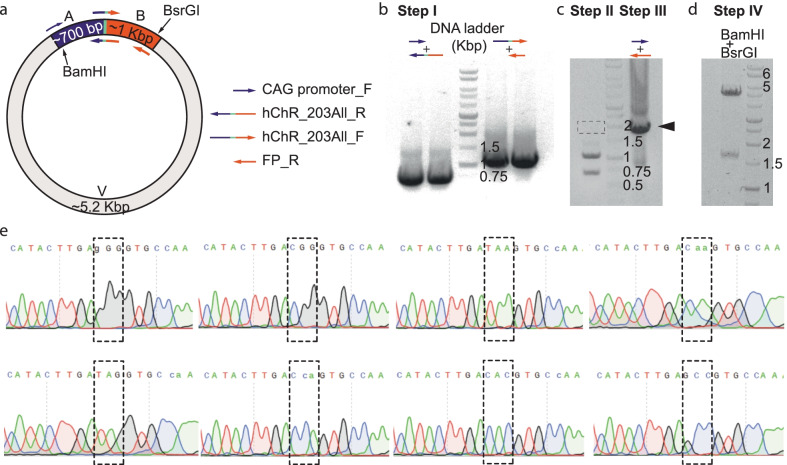
Shuffling of residue M203 of hChR2-mCherry by degenerate primers and by the modified Gibson assembly method. **a** Depiction of the hChR2-mCherry synthetic gene (colored segments, A and B). Fragment A includes sequences spanning from the standard promoter of the plasmid and intracellular linker III (ILIII; purple); fragment B spans from ILIII to the end of the mCherry (orange). Backbone of plasmid is light grey. The degenerate sequence (green) is situated between purple and orange fragments. Sizes (# of bps) of the fragments are noted within the fragments. Intrinsic and unique digestion sites are noted (BamHI and BsrGI). Primers used for amplification of each fragment are noted on the right (with corresponding colors). **b** Image of PCR products from step I on 1% agarose gel. DNA ladder sizes (in Kbp) are noted on the right of ladder. **c** Left lane shows the products of the Gibson assembly prior amplification. Bottom bands show non-assembled fragments. The expected ‘assembled’ product is not detectable (dashed box, ~ 2 Kbp), whereas the amplified assembled Gibson product by CAG promoter_F and the FP_R primers is easily noticeable (right lane; arrowhead). **d** Digested vector by BamHI and BsrGI. **e** Sequences, and matching chromatograms, of DNA sequences obtained from resulting colonies (labeled with dashed box)

We next examined whether we could evolve a single residue within the third intracellular loop of hChR2-mCherry (residue M203). We employed standard SDM degenerate primers to target three bps for evolution (i.e., a mixture of 64 different primers, each 50 bps long; Table 1). Importantly, these primers under standard conditions repeatedly failed in over 20 different trials (Additional file 1: Supplementary text). We then applied our procedure to amplify the DNA flanking the site to be mutated using the degenerate primers separately, combined with sense CAG promoter_F and antisense FP_R primers (Fig. 4a). Standard amplification (1 hr, see Table 3) yielded the expected amplicons (~ 700 and ~ 1040 bps for Part A and B; Fig. 4b, Step I). The product of the Gibson-assembly of the fragments could not be visualized on gel (Fig. 4c; Step II, dashed region) without amplification (Fig. 4c; Step III, arrowhead, ~ 1750 bps).

The next day (following digestion, ligation and bacterial transformation), we isolated DNA from 38 colonies, all of which contained different mutations at the desired site (two colonies contained a mixture of two DNAs), thereby yielding > 95% efficiency (Fig. 4e, dashed regions). Thus, we were able to rapidly evolved residue M203, resulting in a small library of 16 different amino acid substitutions. Interestingly, though beyond the scope of this work, we noted that proline was the most abundant substitution and that none of the colonies contained the original M203 (either from residual template DNA or by mutagenesis) (Additional file 5: Fig. S3).

### Deletion of three residues in ChR2 by re-using primers and a different template DNA

We next assessed the ability of our procedure to exclusively delete three bps (a stop codon) from the hChR2 sequence (employing primers from Fig. 3). We selected one of the clones that was obtained after evolution of hChR2, specifically hChR2-**stop**-R (see Fig. 4). We amplified the two segments of hChR2-stop using the hChR_203_R and _F primer pair in two separate PCR reactions, in combination with the sense CAG promoter_F and the antisense FP_R primers (Additional file 6: Fig. S4a), as previously demonstrated for deletion and replacement of residues in hChR2. This reaction yielded the amplicons of expected sizes (Additional file 6: Fig. S4b), the ligated product could be visualized on gel (Additional file 6: Fig. S4c) and easily ligated into the vector (assessed by digestion analysis, Additional file 6: Fig. S4d). Having consistently obtained very high success rates by our modified approach, we purposefully sequenced isolated DNA from only one colony and, expectedly, find the correct modification in the sequence, namely removal of the stop codon (Additional file 6: Fig. S4e, and compare with Fig. 3d).

## Conclusion

Here, we present a step-by-step protocol for employing the Gibson assembly with non-Gibson primers, rather by re-using standard SDM primers to insert, delete or substitute sequences, whether rationally or randomly (by degenerate primers) within different DNA sequences. In fact, we suggest that the likelihood of having SDM primers in one’s primer-inventory is very high (especially among non-Gibson experts) because, when it comes to mutagenesis, the standard SDM approach is by far the simplest (one primer set), the quickest (one PCR amplification step and products are immediately *dpnI*-digested and transformed into bacteria), the least expensive (PCR reactions are very inexpensive) and most employed. Gibson primers, on the other hand, are more finicky to design [13, 14] (not intuitive to many users), thus less likely present in one’s inventory. Our approach also includes an added PCR-amplification step, as well as includes two optional quality-control steps to reduce uncertainties. More specifically, the amplified Gibson products can be assessed on gel or by DNA sequencing (Fig. 1, step III). The amplified assembled product may also reduce the need to employ additional Gibson reaction mixtures, should the process fail, because the Gibson assembly reaction yields low amounts of product (non-detectable on gel, Fig. 4c) and therefore provide users with limited number of attempts. Thus, amplification of this product provides more product for experimentation. In fact, the ligated product can be repeatedly amplified as necessary. This step allows users to employ higher amounts of amplicons for the ligation step (as suggested in the Gibson assembly protocol for troubleshooting) to increase likelihood of success. Therefore, though optional, the added step may actually reduce time to completion, especially in instances that the process fails after multiple attempts; allowing users to return to the amplified Gibson-ligated products, rather than revert back to earlier reassembly steps (and waste another Gibson reaction mixture).

Together, we present a simple (highly user-friendly), versatile, rapid, and cost-effective modified Gibson assembly approach by use (and re-use) of common SDM primers. We demonstrate that digestion past the entire SDM primer sequence (~ 50 bps) can be obtained within 15 min to yield assembly-*ready* fragments. Our method is of very high efficiency and the final products are obtained quickly (4 days).

## Methods

### Molecular biology and DNA constructs

dLight, hChR2-mCherry were purchased from Addgene (Cat #125,560, Cat #100,054, respectively). Thermocycle (ProFlex, Applied Biosystems) settings and primers employed for amplifications are specifically denoted for each reaction (see Tables 1, 2, 3). Details on standard SDM settings are provided in Additional file 1: Supplementary text. Restriction enzymes (New England Biolabs; NEB) are denoted for each reaction and were incubated with DNA for 1–2 h at 37 °C. 2X KAPA HiFi HotStart ReadyMix (Takara) was used for PCR reactions. Ligations were performed at 18 °C overnight by T4 ligase (NEB). Omni ultracompetent bacterial cells (Zymo) were used for bacterial transformations. DNA purification was performed by use of DNA isolation/purification kit (Zymo).

## Supplementary Information

**Additional file 1:** Supplementary text.

**Additional file 2: Fig. S1.** Illustrations of the modified Gibson assembly method highlighting the end products obtained by the 5′-exonuclease activity at short or long incubation times. Left—Short incubation times may lead to insufficient digestion of DNA and therefore inability to ligate between the fragments (red dashed line at end of process, bottom). Right—Long incubation time and extensive digestion of DNA past the entire sequence of the SDM primer is required to expose overlapping sequences for proper annealing.

**Additional file 3: Fig. S5.** Full length agarose gels for figures 2, 3 and 4 and Additional file 6: Fig. S4. Yellow regions note the relevant lanes for each figure (in cases when multiple amplicons were visualized on the same agarose gel).

**Additional file 4: Fig. S2.** Sequencing of the Gibson assembled fragment after it had undergone amplification and isolation from the agarose gel.

**Additional file 5: Fig. S3.** A small library of hChR2-mCherry variants. Sequences show the translation of the sequencing results obtained from degeneration (scrambling) of residue 204 (yellow highlight). Top-Template DNA used for the reaction.

**Additional file 6: Fig. S4.** Deletion of three residues within hChR2-stop.** a** Depiction of the hChR2-mCherry synthetic gene (colored segments, A and B). Fragment A includes sequences spanning from the standard CAG promoter of the plasmid and intracellular linker III (ILIII; purple); fragment B spans from ILIII to the end of the mCherry (orange). Backbone of plasmid is light grey. Sizes (# of bps) of the fragments are noted within the fragments. Intrinsic and unique digestion sites are also noted (BamHI and BsrGI). Primers used for amplification of each fragment are noted on the right (with corresponding colors). **b** Image of PCR products from step I on 1% agarose gel. DNA ladder sizes (in Kbp) are noted on the right of ladder (white). **c** The amplified assembled Gibson product, obtained by CAG promoter_F and the FP_R primers (purple and orange primers, respectively), visualized on 1% agarose gel. **d** Digested vector is shown on right lane of the right gel (BamHI and BsRGI). **e** Sequence alignment (and matching chromatograms) between template DNA (top) and DNA isolated from a single colony (bottom, dashed box).

## Data Availability

All data are provided in the manuscript and in supplementary material.

## Abbreviations

bp: Base pairs
(K)bp: Base pairs, K—1000
hrs: Hours
SDM: Site directed mutagenesis
FR: Forward (i.e., sense primer)
R: Reverse (i.e., antisense primer)
hChR2-mCherry: Humanized Channelrhodopsin2-mCherry
dLight: Fluorescent (light) Dopamine probe
GFP: Green fluorescent protein
PCR: Polymerase chain reaction (i.e., thermocycle-based reaction)
IL(I–III): Intracellular linker I, II or III.
ddH_2_O: Double distilled water
