# Supplementary material for: A simplified Gibson assembly method for site directed mutagenesis by re-use of standard, and entirely complementary, mutagenesis primers
Source: BMC Biotechnol. 2022 Mar 13;22:10. doi: 10.1186/s12896-022-00740-y (PMC8918331; doi:10.1186/s12896-022-00740-y)
Supplement: Supplementary file 1 — Additional file 1: Supplementary text. [file 12896_2022_740_MOESM1_ESM.docx]

**Supplementary Text**

**Standard thermocycle settings for** **introducing six bps into dLight-GFP—** We employed the QuikChange Lightning Site-Directed Mutagenesis Kit to try to insert the selected codons. The PCR reaction mixture and protocol used are provided in **supplementary tables S1 and S2**, respectively. The PCR products were treated with 1 µl of *Dpn*I at 37°C for 1 hour and then transformed into XL10-Gold ultracompetent cells. No colonies were obtained. Consequently, we doubled the annealing time to 20 sec (**supplementary table S2, Trial 2**), or added 1 µl of DMSO at two different annealing temperatures for 15 seconds (**supplementary tables S1 and S2; Trials 3 and 4**), or decreased annealing temperatures to 55°C and 50°C (**Trials 5 and 6, supplementary table S2**). None proved successful.

**Supplementary Table S1. PCR reaction mixture using QuikChange Lightning kit**

| Component | Trials 1, 2, 5 and 6 | Trials 3 and 4 |
| --- | --- | --- |
| 10× reaction buffer | 2.5 µl | 2.5 µl |
| Template (100 ng) | 1 µl | 1 µl |
| SDM_Primer_F (10 µM stock) | 0.7 µl | 0.7 µl |
| SDM_Primer_R (10 µM stock) | 0.7 µl | 0.7 µl |
| dNTP mix | 0.5 µl | 0.5 µl |
| QuikSolution reagent | 0.75 µl | 0.75 µl |
| DMSO | --- | 1 µl |
| ddH2O | Up to 25 µl | Up to 25 µl |

**Supplementary Table S2. The PCR cycling protocol using QuikChange Lightning kit**

|  | Trial 1 | | Trial 2 | | Trials 3 and 4 | | Trials 5 and 6 | |
| --- | --- | --- | --- | --- | --- | --- | --- | --- |
| Step | **Cycles** | **Temp. / Duration** | **Cycles** | **Temp. / Duration** | **Cycles** | **Temp. / Duration** | **Cycles** | **Temp. / Duration** |
| Initial denaturation | 1 | 95°C /2 minutes | 1 | 95°C /2 minutes | 1 | 95°C /2 minutes | 1 | 95°C /2 minutes |
| Denaturation | 18 | 95°C /20 seconds | 18 | 95°C /20 seconds | 18 | 95°C /20 seconds | 18 | 95°C /20 seconds |
| Annealing |  | 60°C /10 seconds |  | 60°C /20 seconds |  | 58/60°C /15 seconds |  | 50/55°C /10 seconds |
| Extension |  | 68°C /3.5 minutes |  | 68°C /3.5 minutes |  | 68°C /3.5 minutes |  | 68°C /3.5 minutes |
| Final extension | 1 | 68°C /5 minutes | 1 | 68°C /5 minutes | 1 | 68°C /5 minutes | 1 | 68°C /5 minutes |

**Standard thermocycle settings for deletion of six bps and their replacement by three other bps in hChR2-mCherry—** We tried to delete six bps and introduce three bps using the KAPA HiFi HotStart ReadyMix PCR Kit. PCR reaction mixture and protocol used are provided in **supplementary tables S3 and S4**, respectively. The PCR products were treated with 1 µl of *Dpn*I at 37°C for 1 hour and then transformed into Omni ultracompetent cells. The reaction failed to yield any product. We subsequently increased annealing temperatures to 62°C and 67°C (**Trials 2 and 3, supplementary table S4**), yet no colonies were detected following transformation.

**Supplementary Table S3. PCR reaction mixture using KAPA HiFi PCR Kit**

| Component | Trials 1, 2, and 3 |
| --- | --- |
| 2X KAPA HiFi HotStart ReadyMix | 12.5 µl |
| Template (10 ng) | 1 µl |
| SDM_Primer_F (5 µM stock) | 0.75 µl |
| SDM_Primer_R (5 µM stock) | 0.75 µl |
| ddH2O | Up to 25 µl |

**Supplementary Table S4. PCR cycling protocol using KAPA HiFi HotStart ReadyMix PCR Kit**

|  | Trials 1-3 | |
| --- | --- | --- |
| Step | Cycles | Temperature/ Duration |
| Initial denaturation | 1 | 95°C /3 minutes |
| Denaturation | 20 | 98°C /20 seconds |
| Annealing |  | 67/62/60°C /20 seconds |
| Extension |  | 72°C /7 minutes |
| Final extension | 1 | 72°C /7 minutes |

Next, we employed QuickChange Lightning Site-Directed Mutagenesis Kit. Standard PCR reaction mixture was used (**supplementary table S1, Trial 1**), with 20 amplification cycles, and annealing at 55°C or 60°C for 20 seconds. No colonies were detected following the transformation into the XL10-Gold ultracompetent cells.

**Standard thermocycle settings for degeneration of residue M203 in hChR2—** We employed the QuickChange Lightning Site-Directed Mutagenesis Kit to substitute residue 203. Standard PCR reaction mixture (and protocols were used (**supplementary tables S5 and S2**). No colonies were obtained following transformation and growth overnight. Consequently, we decreased the temperature of the annealing to 55°C and even to 50°C, while increasing annealing time to 45 seconds (**Trials 2 and 3**) with no success.

We proceeded to examine touchdown and touchup PCR protocols, in which the annealing temperature decreases or increases, respectively. We used the QuickChange Lightning Site-Directed Mutagenesis Kit, in two separate reactions provided in **supplementary table S6** (**Trials 4 and 5**). Following *Dpn*I treatment and transformation, we obtained few colonies only by the touchdown protocol, but all colonies were negative (verified by DNA sequencing). Increased or decrease in primer concentrations with touchdown PCR failed to yield any colonies (**supplementary tables S5 and S6, Trial 7**; **supplementary table S5, Trial 8, respectively**).

**Supplementary Table S5. The reaction ingredients to substitutes hChR2 203M using QuikChange Lightning kit**

| Component | Trials 1-5 | Trials 6 and 7 | Trial 8 |
| --- | --- | --- | --- |
| 10× reaction buffer | 2.5 µl | 2.5 µl | 2.5 µl |
| Template (100 ng) | 1 µl | 1 µl | 1 µl |
| SDM_Primer_F | 0.7 µl (10 µM) | 0.7 µl (100 µM) | 0.7 µl (1 µM) |
| SDM_Primer_R | 0.7 µl (10 µM) | 0.7 µl (100 µM) | 0.7 µl (1 µM) |
| dNTP mix | 0.5 µl | 0.5 µl | 0.5 µl |
| QuikSolution reagent | 0.75 µl | 0.75 µl | 0.75 µl |
| ddH2O | Up to 25 µl | Up to 25 µl | Up to 25 µl |

**Supplementary Table S6. Touch-down and touch-up PCR cycling protocol using QuikChange Lightning kit**

|  | Trials 4 and 6 | | Trials 5 and 7 | |
| --- | --- | --- | --- | --- |
| Step | Cycles | Temperature/ Duration | Cycles | Temperature/ Duration |
| Initial denaturation | 1 | 95°C /2 minutes | 1 | 95°C /2 minutes |
| Denaturation | 30 | 95°C /20 seconds | 30 | 95°C /20 seconds |
| Annealing |  | 60°C (-1°C)* /20 seconds |  | 30°C (+1°C)* /20 seconds |
| Extension |  | 68°C /3.5 minutes |  | 68°C /3.5 minutes |
| Denaturation | 20 | 95°C /20 seconds | 20 | 95°C /20 seconds |
| Annealing |  | 55°C /20 seconds |  | 55°C /20 seconds |
| Extension |  | 68°C /3.5 minutes |  | 68°C /3.5 minutes |
| Final extension | 1 | 68°C /5 minutes | 1 | 68°C /5 minutes |

* decreasing/ increasing in 1°C every cycle

We next used KAPA HiFi HotStart ReadyMix PCR Kit. PCR reaction mixture matched the manufacturer’s recommendations (**supplementary table S3**). We adjusted the annealing time to 15 sec at 60°C (**Trial 9**). PCR products treated with 1 µl of *Dpn*I at 37°C for 1 hour were transformed in Omni ultracompetent cells, yet no colonies grew. Touchdown PCR with this kit (**supplementary table S7**, **Trial 10**) also yielded no results, even when we extensively varied the annealing temperatures (**supplementary table S7, Trials 11-14**).

**Supplementary Table S7. Touch-down and touch-up PCR cycling protocol using KAPA HiFi**

|  | Trial 10 | | Trials 11 and 12 | | Trials 13 and 14 | |
| --- | --- | --- | --- | --- | --- | --- |
| Step | Cycles | Temperature/ Duration | Cycles | Temperature/ Duration | Cycles | Temperature/ Duration |
| Initial denaturation | 1 | 95°C /3 minutes | 1 | 95°C /3 minutes | 1 | 95°C /3 minutes |
| Denaturation | 35 | 98°C /25 seconds | 40 | 98°C /25 seconds | 30 | 98°C /20 seconds |
| Annealing |  | 60°C (-0.4°C)* /1 minute |  | 60°C/55°C (-0.5°C)* /1 minute |  | 60°C/ 30°C (-/+1°C)* /30 seconds |
| Extension |  | 72°C /6 minutes |  | 72°C /6 minutes |  | 72°C /6 minutes |
| Denaturation | 25 | 98°C /20 seconds | 25 | 98°C /25 seconds | 25 | 98°C /20 seconds |
| Annealing |  | 50°C /1 minute |  | 55°C/50°C  /1 minute |  | 50°C /30 seconds |
| Extension |  | 72°C /6 minutes |  | 72°C /6 minutes |  | 72°C /6 minutes |
| Final extension | 1 | 72°C /15 minutes | 1 | 72°C /15 minutes | 1 | 72°C /15 minutes |

* decreasing/ increasing in X°C every cycle
