# Supplementary figures and images for: A simplified Gibson assembly method for site directed mutagenesis by re-use of standard, and entirely complementary, mutagenesis primers
Source: BMC Biotechnol. 2022 Mar 13;22:10. doi: 10.1186/s12896-022-00740-y (PMC8918331; doi:10.1186/s12896-022-00740-y)

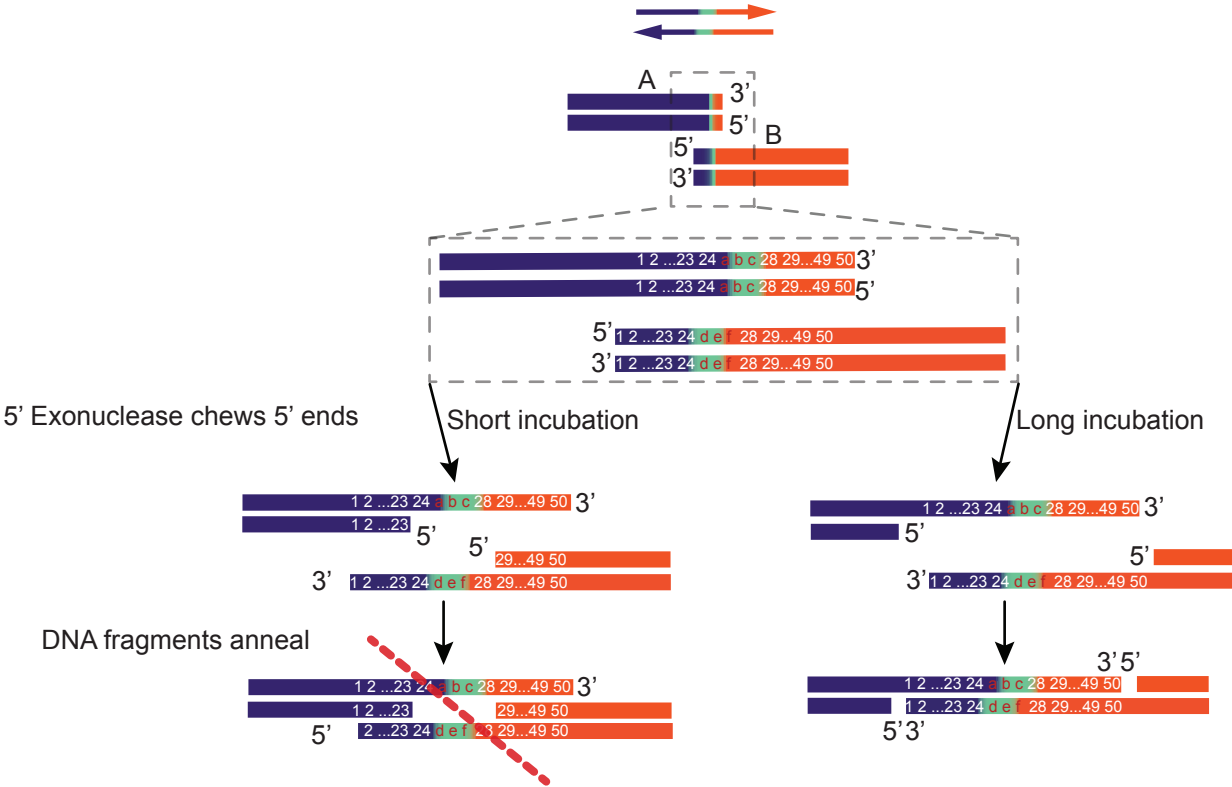

Supplement: Supplementary file 2 — Additional file 2: Fig. S1. Illustrations of the modified Gibson assembly method highlighting the end products obtained by the 5′-exonuclease activity at short or long incubation times. Left—Short incubation times may lead to insufficient digestion of DNA and therefore inability to ligate between the fragments (red dashed line at end of process, bottom). Right—Long incubation time and extensive digestion of DNA past the entire sequence of the SDM primer is required to expose overlapping sequences for proper annealing. [file 12896_2022_740_MOESM2_ESM.pdf]

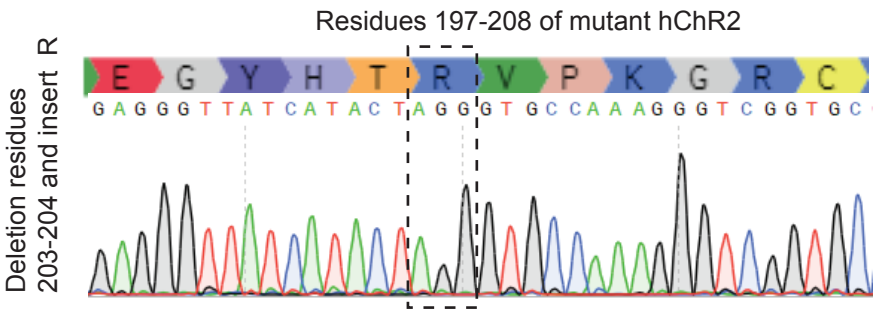

Supplement: Supplementary file 4 — Additional file 4: Fig. S2. Sequencing of the Gibson assembled fragment after it had undergone amplification and isolation from the agarose gel. [file 12896_2022_740_MOESM4_ESM.pdf]

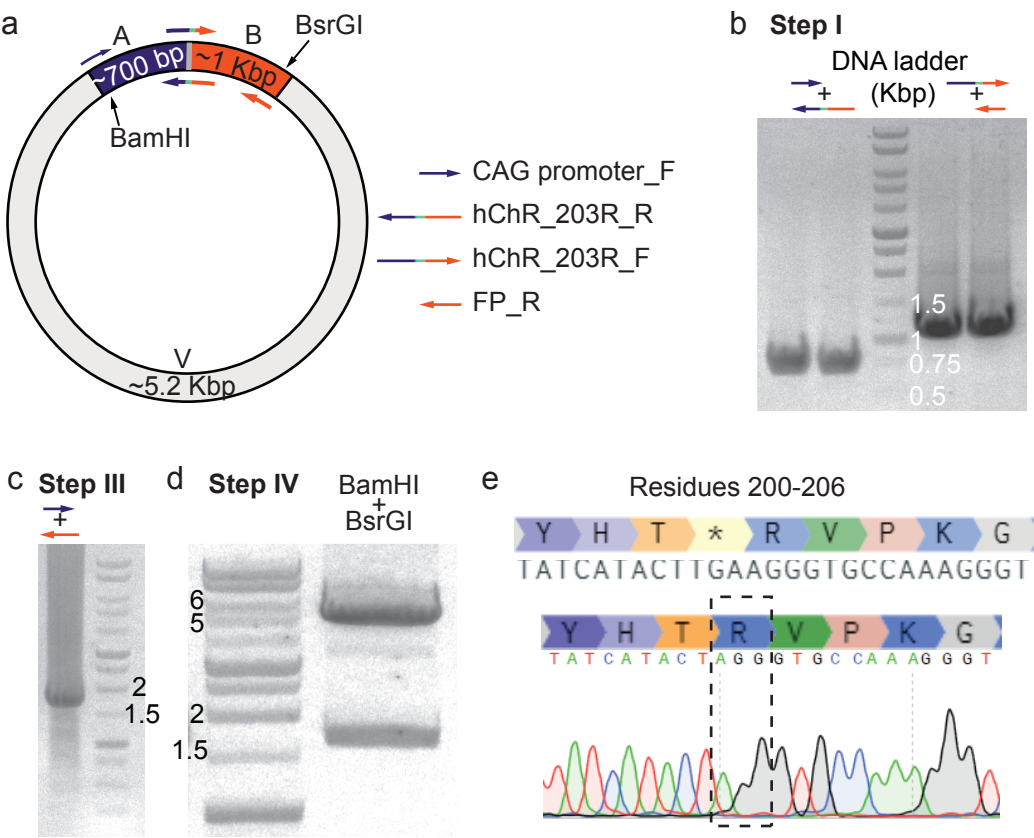

Supplement: Supplementary file 6 — Additional file 6: Fig. S4. Deletion of three residues within hChR2-stop. a Depiction of the hChR2-mCherry synthetic gene (colored segments, A and B). Fragment A includes sequences spanning from the standard CAG promoter of the plasmid and intracellular linker III (ILIII; purple); fragment B spans from ILIII to the end of the mCherry (orange). Backbone of plasmid is light grey. Sizes (# of bps) of the fragments are noted within the fragments. Intrinsic and unique digestion sites are also noted (BamHI and BsrGI). Primers used for amplification of each fragment are noted on the right (with corresponding colors). b Image of PCR products from step I on 1% agarose gel. DNA ladder sizes (in Kbp) are noted on the right of ladder (white). c The amplified assembled Gibson product, obtained by CAG promoter_F and the FP_R primers (purple and orange primers, respectively), visualized on 1% agarose gel. d Digested vector is shown on right lane of the right gel (BamHI and BsRGI). e Sequence alignment (and matching chromatograms) between template DNA (top) and DNA isolated from a single colony (bottom, dashed box). [file 12896_2022_740_MOESM6_ESM.pdf]
