# Supplementary material for: A simplified Gibson assembly method for site directed mutagenesis by re-use of standard, and entirely complementary, mutagenesis primers
Source: BMC Biotechnol. 2022 Mar 13;22:10. doi: 10.1186/s12896-022-00740-y (PMC8918331; doi:10.1186/s12896-022-00740-y)

Olszakier et la. Supplementary Figure S5.

Figure 2 panel b

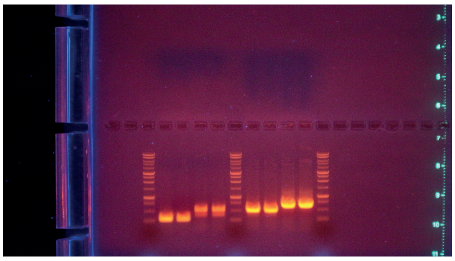

Figure 2 panel c

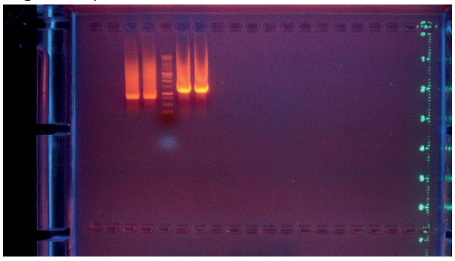

Figure 3 panel b

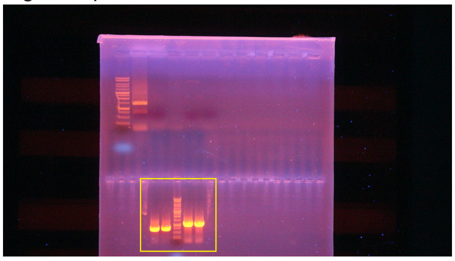

Figure 3 panel c (left)

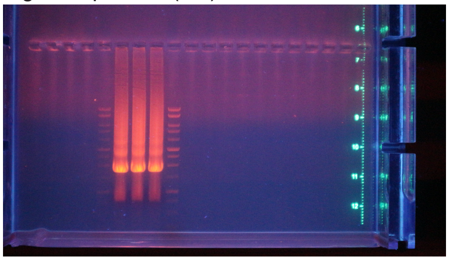

Figure 3 panel c (right)

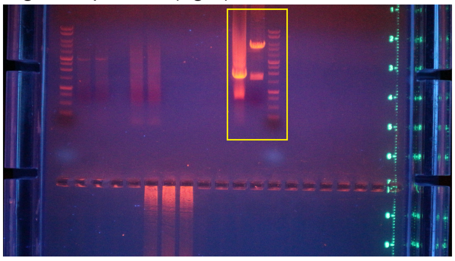

Figure 4 panel b

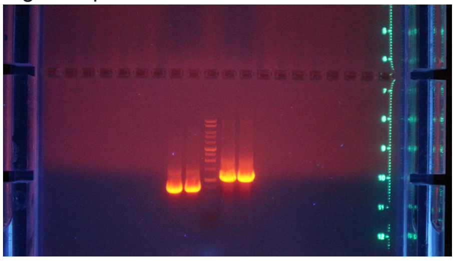

Figure 4 panel c

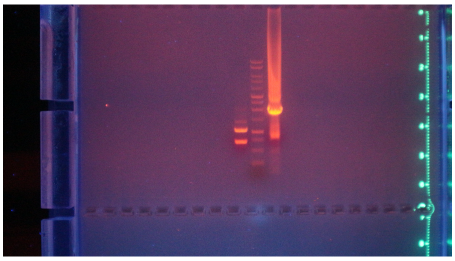

Figure 4 panel d

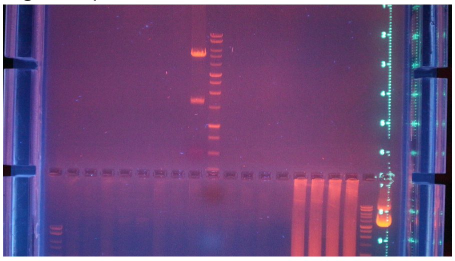

Supplementary Figure S4 panel b

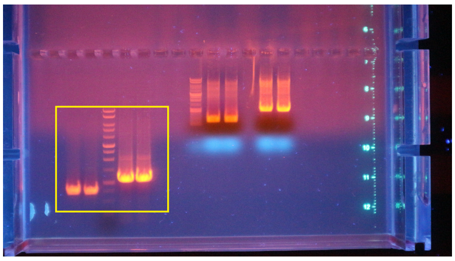

Supplementary Figure S4 panel c

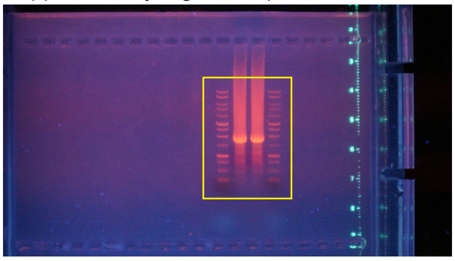

Supplementary Figure S4 panel d

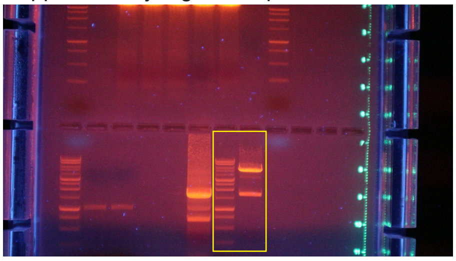

Supplement: Supplementary file 3 — Additional file 3: Fig. S5. Full length agarose gels for figures 2, 3 and 4 and Additional file 6: Fig. S4. Yellow regions note the relevant lanes for each figure (in cases when multiple amplicons were visualized on the same agarose gel). [file 12896_2022_740_MOESM3_ESM.pdf]
