# Supplementary material for: A simplified Gibson assembly method for site directed mutagenesis by re-use of standard, and entirely complementary, mutagenesis primers
Source: BMC Biotechnol. 2022 Mar 13;22:10. doi: 10.1186/s12896-022-00740-y (PMC8918331; doi:10.1186/s12896-022-00740-y)
Supplement: Supplementary file 5 — Additional file 5: Fig. S3. A small library of hChR2-mCherry variants. Sequences show the translation of the sequencing results obtained from degeneration (scrambling) of residue 204 (yellow highlight). Top-Template DNA used for the reaction. [file 12896_2022_740_MOESM5_ESM.pdf]

Olszakier et al. Supplementary Figure S3

hChR2 residues 176-229

	Template	IFFCLGLCYGANTFFHAAKAYIEGYHT*MVPKGRCRQVVTGMAWLFFVSWGMFPIL
X2	A [GCC]	IFFCLGLCYGANTFFHAAKAYIEGYHT*AVPKGRCRQVVTGMAWLFFVSWGMFPIL
	C [TGC]	IFFCLGLCYGANTFFHAAKAYIEGYHT*CVPKGRCRQVVTGMAWLFFVSWGMFPIL
	C [TGT]	IFFCLGLCYGANTFFHAAKAYIEGYHT*CVPKGRCRQVVTGMAWLFFVSWGMFPIL
	C [TAT]	IFFCLGLCYGANTFFHAAKAYIEGYHT*CVPKGRCRQVVTGMAWLFFVSWGMFPIL
	E [GAA]	IFFCLGLCYGANTFFHAAKAYIEGYHT*EVPKGRCRQVVTGMAWLFFVSWGMFPIL
	G [GGG]	IFFCLGLCYGANTFFHAAKAYIEGYHT*GVPKGRCRQVVTGMAWLFFVSWGMFPIL
	H [CAC]	IFFCLGLCYGANTFFHAAKAYIEGYHT*HVPKGRCRQVVTGMAWLFFVSWGMFPIL
	I [ATC]	IFFCLGLCYGANTFFHAAKAYIEGYHT*IVPKGRCRQVVTGMAWLFFVSWGMFPIL
	L [CTG]	IFFCLGLCYGANTFFHAAKAYIEGYHT*LVPKGRCRQVVTGMAWLFFVSWGMFPIL
	L [TTG]	IFFCLGLCYGANTFFHAAKAYIEGYHT*LVPKGRCRQVVTGMAWLFFVSWGMFPIL
	L [TTA]	IFFCLGLCYGANTFFHAAKAYIEGYHT*LVPKGRCRQVVTGMAWLFFVSWGMFPIL
	N [AAT]	IFFCLGLCYGANTFFHAAKAYIEGYHT*NVPKGRCRQVVTGMAWLFFVSWGMFPIL
X3	P [CCA]	IFFCLGLCYGANTFFHAAKAYIEGYHT*PVPKGRCRQVVTGMAWLFFVSWGMFPIL
X2	P [CCC]	IFFCLGLCYGANTFFHAAKAYIEGYHT*PVPKGRCRQVVTGMAWLFFVSWGMFPIL
X3	P [CCG]	IFFCLGLCYGANTFFHAAKAYIEGYHT*PVPKGRCRQVVTGMAWLFFVSWGMFPIL
	Q [CAA]	IFFCLGLCYGANTFFHAAKAYIEGYHT*QVPKGRCRQVVTGMAWLFFVSWGMFPIL
	R [AGG]	IFFCLGLCYGANTFFHAAKAYIEGYHT*RVPKGRCRQVVTGMAWLFFVSWGMFPIL
X3	R [CGG]	IFFCLGLCYGANTFFHAAKAYIEGYHT*RVPKGRCRQVVTGMAWLFFVSWGMFPIL
	S [AGC]	IFFCLGLCYGANTFFHAAKAYIEGYHT*SVPKGRCRQVVTGMAWLFFVSWGMFPIL
	S [TCC]	IFFCLGLCYGANTFFHAAKAYIEGYHT*SVPKGRCRQVVTGMAWLFFVSWGMFPIL
	S [TCA]	IFFCLGLCYGANTFFHAAKAYIEGYHT*SVPKGRCRQVVTGMAWLFFVSWGMFPIL
	T [ACC]	IFFCLGLCYGANTFFHAAKAYIEGYHT*TVPKGRCRQVVTGMAWLFFVSWGMFPIL
	V [GTG]	IFFCLGLCYGANTFFHAAKAYIEGYHT*VVPKGRCRQVVTGMAWLFFVSWGMFPIL
	V [GTT]	IFFCLGLCYGANTFFHAAKAYIEGYHT*VVPKGRCRQVVTGMAWLFFVSWGMFPIL
	W [TGG]	IFFCLGLCYGANTFFHAAKAYIEGYHT*WVPKGRCRQVVTGMAWLFFVSWGMFPIL
	Y [TAC]	IFFCLGLCYGANTFFHAAKAYIEGYHT*YVPKGRCRQVVTGMAWLFFVSWGMFPIL
	* [TAG]	IFFCLGLCYGANTFFHAAKAYIEGYHT**VPKGRCRQVVTGMAWLFFVSWGMFPIL
	* [TAA]	IFFCLGLCYGANTFFHAAKAYIEGYHT**VPKGRCRQVVTGMAWLFFVSWGMFPIL
